# Mouse, but Not Human, ApoB-100 Lipoprotein Cholesterol Is a Potent Innate Inhibitor of *Streptococcus pneumoniae* Pneumolysin

**DOI:** 10.1371/journal.ppat.1004353

**Published:** 2014-09-04

**Authors:** Kristin R. Wade, Eileen M. Hotze, David E. Briles, Rodney K. Tweten

**Affiliations:** 1 Department of Microbiology and Immunology, The University of Oklahoma Sciences Center, Oklahoma City, Oklahoma, United States of America; 2 Department of Microbiology, University of Alabama at Birmingham, Birmingham, Alabama, United States of America; Harvard Medical School, United States of America

## Abstract

*Streptococcus pneumoniae* produces the pore-forming toxin pneumolysin (PLY), which is a member of the cholesterol-dependent cytolysin (CDC) family of toxins. The CDCs recognize and bind the 3β-hydroxyl group of cholesterol at the cell surface, which initiates membrane pore formation. The cholesterol transport lipoproteins, which carry cholesterol in their outer monolayer, are potential off-pathway binding targets for the CDCs and are present at significant levels in the serum and the interstitial spaces of cells. Herein we show that cholesterol carried specifically by the ApoB-100-containing lipoprotein particles (CH-ApoB-100) in the mouse, but not that carried by human or guinea pig particles, is a potent inhibitor of the PLY pore-forming mechanism. Cholesterol present in the outer monolayer of mouse ApoB-100 particles is recognized and bound by PLY, which stimulates premature assembly of the PLY oligomeric complex thereby inactivating PLY. These studies further suggest that the vast difference in the inhibitory capacity of mouse CH-ApoB-100 and that of the human and the guinea pig is due to differences in the presentation of cholesterol in the outer monolayer of their ApoB-100 particles. Therefore mouse CH-ApoB-100 represents a significant innate CDC inhibitor that is absent in humans, which may underestimate the contribution of CDCs to human disease when utilizing mouse models of disease.

## Author Summary

The pore-forming cholesterol-dependent cytolysins (CDCs) are one of the most widely disseminated virulence factors expressed by Gram-positive pathogens. *Streptococcus pneumoniae* is a major human pathogen and expresses a CDC termed pneumolysin (PLY). PLY and most CDCs bind cholesterol as their cellular receptor, which initiates the formation of the oligomeric pore complex. Our studies show the cholesterol carried by mouse ApoB-100 (CH-ApoB-100), but not human or guinea pig ApoB-100 lipoproteins, acts as a potent innate PLY inhibitor. This selective inhibitory capacity is not due to differences in CH-ApoB-100 levels, but appears to result from differences in cholesterol presentation at the surface of the ApoB-100 particle from these species. Our results suggest bacterial pathogenesis studies of *S. pneumoniae* and other CDC-producing bacteria utilizing mouse animal models may not reflect the CDCs true contribution to human disease or the potential efficacy of CDC-based vaccines due to the innate potent CDC inhibitory activity of mouse CH-ApoB-100.

## Introduction

**Figure 1:**
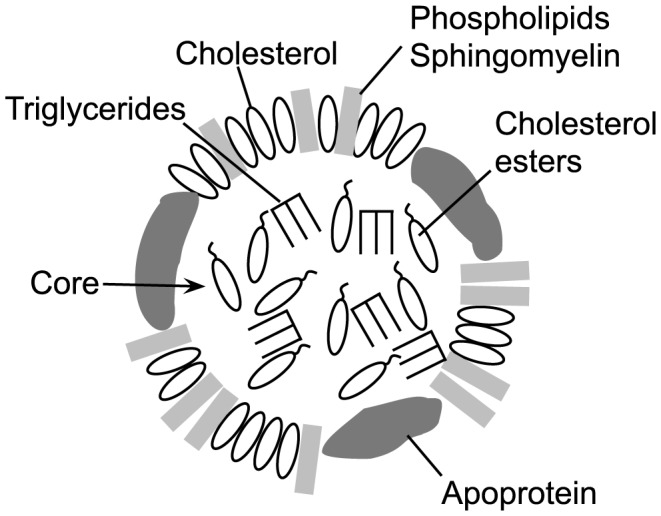
Schematic representation of an LDL or HDL lipoprotein particle. A typical lipoprotein particle is shown (not to scale), featuring an outer monolayer consisting of phospholipids, sphingomyelin and cholesterol. The particle core contains mainly triglycerides and cholesterol esters, although some cholesterol may be present. The associated apoprotein is shown in the outer monolayer, although it can also traverse the core. The apoprotein(s) present is (are) specific to the lipoprotein particle type [2], [19].

A major component of the mammalian cellular membrane is cholesterol, which is transported to and from cells via lipoprotein cholesterol carriers [1], [2]. Membrane cholesterol serves as the receptor for most cholesterol-dependent cytolysins (CDCs), which contribute to pathogenesis in a wide variety of Gram-positive bacterial pathogens (reviewed in [3]). Cholesterol binding is mediated via a strictly conserved Thr-Leu cholesterol-recognition motif (CRM) in domain 4 of the CDC structure [4]. The CRM specifically recognizes the cholesterol 3β-hydroxyl group: modifications to this group render cholesterol inert to CDC recognition [5], [6]. Cholesterol binding then initiates the formation of the CDC oligomeric pore complex [7]. In addition to cellular membranes, cholesterol is also located in the outer lipid monolayer shell and core of lipoprotein particles (Figure 1), which are found in abundance in the serum, lymph and interstitial spaces. Therefore, the cholesterol carried by these particles represents a potential off-pathway target for the CDCs, which could lead to their inactivation.

Classically, CDC inactivation with pure cholesterol micelles has been used as one method to confirm the identity of putative CDCs [8]–[13]. The basis for this potent CDC inhibition was shown by Heuck et al. [14] to result from micellar cholesterol-induced formation of the CDC oligomeric pore complex, which cannot then interact with cells. Importantly, cholesterol micelles are a monolayer, thus showing that cholesterol does not have to be packaged in a bilayer structure to serve as a receptor for CDCs. In an analogous fashion to cholesterol micelles, lipoprotein particles maintain cholesterol in their outer monolayer with a number of lipids (Figure 1) and thus could be recognized and bound by CDCs. It is also important to note that the CDCs only bind a small fraction of the total available cholesterol in a membrane [15]. The lipid environment of the cholesterol is a major determinant of its availability for CRM-mediated binding [16]–[18]. Lipids that tend to pack tightly or have a large headgroup significantly decrease CDC recognition and binding to cholesterol, whereas lipids that pack loosely with cholesterol or have small headgroups promote binding [17], [18]. Therefore, the outer monolayer lipid structure surrounding exposed cholesterol on lipoproteins could also impact the ability of the CDCs to bind cholesterol.

Cholesterol is carried throughout the body by lipoprotein particles such as HDL (high density lipoproteins), LDL (low density lipoproteins), IDL (intermediate density lipoproteins), VLDL (very low density lipoproteins) and chylomicrons. The lipoprotein core of LDL and HDL (Figure 1) primarily contains esterified cholesterol (cholesterol with a 3β-hydroxyl modified with fatty acids) and triglycerides, which is enclosed by an outer lipid monolayer surface composed primarily of sphingomyelin, phosphatidylcholine, cholesterol, cholesterol ester and the particle specific apolipoprotein(s) (reviewed in [19]). Chylomicrons contain several lipoproteins including apolipoprotein A1 (ApoA1), apolipoprotein B-100 (ApoB-100), apolipoprotein C (ApoC) and apolipoprotein E (ApoE), while HDL is primarily associated with ApoA1, and VLDL, IDL and LDL are associated with ApoB-100 or ApoB-48 (a cleavage product of ApoB-100) [20].

Many human commensal and opportunistic pathogens express a CDC, including *Streptococcus pneumoniae*, which produces the CDC pneumolysin (PLY) [21]. PLY is released into the extracellular milieu by an unidentified mechanism [22]–[24], where PLY can interact with host cells and serum components resulting in pore formation, induction of apoptosis and complement activation (reviewed in [25]). Mammalian cholesterol transport lipoproteins present in the host tissues constitute potential off-pathway binding targets for PLY and others CDCs that could result in the inactivation of their pore-forming activity.

Herein we reveal that the cholesterol carried by mouse ApoB-100 (CH-ApoB-100) but not ApoA1 lipoprotein particles is a potent inhibitor of CDC lytic activity. In striking contrast, human and guinea pig lipoproteins were not found to be significantly inhibitory to the CDCs. PLY neutralization by mouse CH-ApoB-100 results from cholesterol stimulated binding and oligomer formation, which prevents subsequent pore formation in cell membranes. Furthermore, the data strongly suggest the basis for the vast difference in the inhibitory activity of mouse and human lipoproteins is due to the presentation of cholesterol on the surface of the lipoprotein lipid monolayer, rather than the absolute cholesterol concentration carried by these particles. Hence, mice contain a potent innate PLY inhibitor that is largely absent in humans. Therefore, the contribution of PLY and other CDCs to pathogenesis in human infections, as well as the efficacy of CDC-based vaccines, may be underestimated in mouse models.

## Results

### Serum inhibition of CDC lytic activity

The ability of non-immune serum to inhibit PLY hemolytic activity was first examined using pooled mouse (non-Swiss albino) and human sera. Mouse serum inhibited PLY hemolytic activity >3,000-fold, compared to a 62-fold inhibition in the presence of human serum (Table 1). We should note the inhibitory activity of these sera vary from lot-to-lot, but does not vary by more than approximately 30% in our experience. Diurnal variation in mouse cholesterol levels due to feeding is typically less than 2-fold [26].

**Table 1:** Inhibitory activity of mouse, human and guinea pig sera.

Diluent	PLY	PFO	SLO
PBS	1.5±0.1	0.3±0.02	5±0.2
Mouse serum	4,720±710 (**3,147**)	98±0.71 (**327**)	1,410±19 (**282**)
PBS	1.4±0.1	0.3±0.06	4±0.6
Human serum	87±0.7 (62)	5±0.1 (7)	380±0.1 (95)*
PBS	0.5±0.1	2±0.03	4±0.2
Guinea pig serum	13±0.6 (26)	3±1 (1.5)	7±1 (2)

Shown are the average EC_50_ values, standard deviation (SD) and fold-change in EC_50_ for PLY, PFO and SLO when treated with sera from mice, humans and guinea pigs compared to the EC_50_ obtained in the absence of sera (toxin sample is serially diluted in PBS or sera in PBS). Each sera tested was matched to its own individual PBS positive control. The fold change  = EC_50_ in the presence of serum/EC_50_ in PBS, and is shown in parenthesis. Results from 3 independent experiments are shown.

*Inhibition of SLO by human serum likely due to high SLO antibody titers, which were 47,000 to 128,000, depending on the serum lot.

Mouse and human sera contain complement proteins that could potentially affect the hemolytic assay and an interaction between PLY and complement component C1q has been suggested to deplete complement during infection [27]–[30]. Therefore, any potential for a direct or indirect complement effect on PLY-mediated hemolysis activity was investigated by repeating these experiments using sera in which the complement had been heat-inactivated. Heat-inactivation had no significant effect on serum-dependent inhibition of PLY hemolytic activity compared to unheated controls (data not shown).

To determine if sera from other strains of mice commonly used in *S. pneumoniae* studies also inhibited PLY, serum samples were obtained from CBA/N/CaHN-Btkxid/J, BALB/c and C57BL/6 mice and compared to the inhibition observed using serum from non-Swiss albino mice (note, the sublines for the BALB/c, C57BL/6 and non-Swiss albino were not available). Sera samples from these mouse strains exhibited significant PLY inhibitory activity that varied considerably among the strains, but was consistently high (Table 2). This observation suggests that sera from mice contain somewhat variable levels of a potent PLY inhibitor. To understand the basis for the significant difference in the inhibitory activity of mouse versus human serum the remainder of the studies used non-Swiss albino serum.

**Table 2:** Inhibition of PLY hemolytic activity by serum from different mouse strains.

Buffer or Mouse strain	EC_50_ (nM)	EC_50_ serum/EC_50_ PBS
PBS	0.09±0.02	
Non-Swiss Albino	420±10	4,667
CBA/N	64±2	711
BALB/c	1230±110	13,667
C57BL/6	1670±40	18,556

Shown are the average EC_50_ values, SD and fold-change in EC_50_ of PLY-mediated hemolysis of sheep RBCs after serial dilution into PBS containing 10% sera from the indicated mouse strains. The fold change  = EC_50_ in the presence of serum/EC_50_ in PBS. Results from 3 independent experiments are shown. No PLY antibodies were detected in these sera (see Table S1). Note: Non-Swiss albino serum for Table 1 was from a different pooled lot as that shown here.

We also determined whether human and mouse sera exhibited similar abilities to inhibit other members of the CDC family [7]. Sera were incubated with the CDCs from *Clostridium perfringens*, perfringolysin O (PFO), and *Streptococcus pyogenes*, streptolysin O (SLO). The non-Swiss albino mouse serum inhibited PFO and SLO less efficiently than PLY (Table 1). Human serum inhibited PFO less efficiently than PLY, but inhibited SLO more efficiently than PLY (Table 1). However, as will be shown below, the majority of the PLY inhibitory activity in human serum was due to PLY antibodies. The majority of the inhibitory activity against SLO present in the pooled human sera was likely due to SLO antibodies, as antibodies to this CDC are commonly found in humans [31], [32]. SLO antibodies were found in the pooled human sera tested herein at titers >40,000 (data not shown), a fraction of which were likely to neutralize SLO activity.

We also examined the inhibitory activity of guinea pig sera against the lytic activity of SLO, PFO and PLY because guinea pigs have been used as a model of *S. pneumoniae* disease [33]–[38]. As can be seen in Table 1 guinea pig serum was relatively ineffective in inhibiting both PFO and SLO lytic activity, and was only slightly better at inhibiting PLY.

### Neutralizing antibody to PLY hemolytic activity in human, mouse and guinea pig sera

Upon exposure to *S. pneumoniae* or PLY alone, a fraction of the total PLY-specific antibody produced is neutralizing to PLY pore-forming activity [39], [40]. An ELISA assay was initially performed to identify the presence of PLY antibodies in our various sera samples. Low to moderate levels of PLY antibodies were present in different lots of guinea pig and human sera, but were not detectable in any of the sera from the different mouse strains (Table S1). Differences in PLY antibody titers in human or guinea pig sera from lot-to-lot caused slight variation in the results of some experiments utilizing sera from these two species.

To evaluate the extent that PLY antibodies in guinea pig and human sera contributed to the observed PLY inhibition, PLY antibodies were removed from human and guinea pig sera by passing sera over an affinity column coupled with PLY, which removed more than 94% of the PLY antibodies (Figure S1). PLY antibody depleted or untreated serum were then incubated with native PLY for 20 minutes at 37°C and the extent of PLY-mediated hemolytic activity inhibition determined. PLY antibody depletion reduced the neutralization capacity of the untreated human serum by 30-fold and guinea pig serum by 40-fold (Table 3). Therefore PLY neutralizing antibodies are the dominant contributor to the PLY inhibition exhibited by human and guinea pig sera, as antibody removal nearly abolished the PLY inhibition observed with the untreated sera. Hence, both guinea pig and human sera exhibit little innate ability to inhibit PLY when compared to the mouse serum.

**Table 3:** Inhibitory activity of human and guinea pig sera depleted of PLY antibodies.

Buffer or Serum	EC_50_ (µM)	EC_50_ serum/EC_50_ PBS
PBS	0.06±0.03	
Human (untreated)	29±6	483
Anti-PLY depleted	0.9±0.4	15
Guinea pig (untreated)	5±1	83
Anti-PLY depleted	0.1±0.06	2

Shown are the average EC_50_ values, SD and fold-change in EC_50_ values for PLY treated with complete human or guinea pig sera or PLY-antibody depleted sera. The fold change  = EC_50_ in the presence of serum/EC_50_ in PBS. Results from 3 independent assays are shown. Note that PLY antibody titers can vary in various lots of serum: hence, the sera analyzed for antibody here are different lots than those used in Table 1. This human serum sample displayed a higher anti-PLY titer than that shown in Table 1 (refer to Table S1).

### PLY susceptibility to serum proteases

Human and mouse sera are known to contain proteases that cleave anthrax protective antigen and lethal toxin, thus preventing toxin-mediated damage [41], [42]. The capacity of the sera proteases herein to cleave PLY and thus inhibit PLY activity was tested by utilizing a derivative of PLY that is deficient in cholesterol binding, PLY^CRM^, due to a Leu-460 substitution for an aspartate of the cholesterol recognition motif (CRM), as formation of the oligomeric complex is inhibited in this mutant [4], [43]. A cysteine was substituted into PLY^CRM^ for Asp-117, which was then purified and the cysteine sulfhydryl modified with a fluorescent dye (Alexa Fluor 488). Fluorescent PLY^CRM^ was then incubated with mouse or human sera for one hour at 37°C. Samples were taken at various times, separated by SDS gel electrophoresis and the fluorescence emission of the Alexa labeled PLY^CRM^ band was analyzed to identify PLY degradation products. PLY^CRM^ cleavage was not observed in the presence of either mouse or human sera over a period of one hour (Figure S2).

### Cholesterol with an intact 3β-hydroxyl is necessary for PLY inhibition

**Figure 2:**
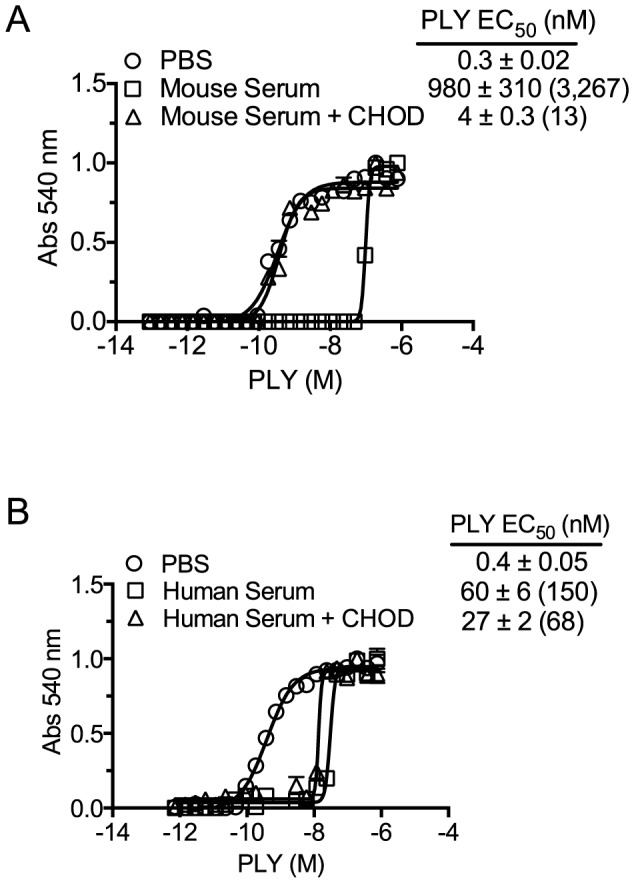
Serum inhibition of PLY requires cholesterol with a free 3β-hydroxyl. PLY requires the free 3β-hydroxyl of cholesterol to bind therefore treatment with CHOD will abolish PLY-cholesterol binding. Samples containing PBS, PBS +10% mouse serum or PBS +50% human serum were incubated with and without cholesterol oxidase (CHOD) for 30 minutes at room temperature to oxidize the 3β-hydroxyl. PLY (38 nmol/150 µl) was then serially diluted using untreated or CHOD-treated mouse (A) or human sera (B) and incubated 20′ at 37°C before RBC hemolysis was determined. The EC_50_ values (toxin concentration required to lyse 50% of RBCs) and the standard deviations (SD) are shown for each sample. The fold difference in the EC_50_ for CHOD treated and untreated samples versus PLY in the absence of serum (PBS control) is shown in parenthesis. Assays were done in duplicate; the data represent three independent experiments. The EC_50_ calculation included the starting serum dilutions (mouse serum was initially diluted to a greater extent, as the mouse serum was found to be significantly more inhibitory than human serum).

The above studies demonstrated that neither antibody to PLY nor proteases in mouse serum were responsible for its ability to inhibit PLY. If cholesterol carried by the lipoproteins was responsible for the inhibition of PLY hemolytic activity then enzymatic treatment of the sera with cholesterol oxidase (CHOD) would decrease or eliminate this inhibitory activity by oxidizing the 3β-hydroxyl thus converting cholesterol to 4-cholesten-3-one, which is not recognized by CDCs [5], [6]. The PLY inhibitory capacity of mouse serum treated with CHOD was reduced by >99.5% compared to untreated serum (Figure 2A). CHOD treatment of human serum had only a slight effect on its ability to inhibit PLY-mediated hemolysis (Figure 2B) consistent with results in Table 3 that showed the majority of the inhibitory activity in human serum was due to PLY neutralizing antibodies. These data show that cholesterol with a free 3β-hydroxyl is the primary component of mouse serum that inhibits PLY.

### Mouse serum induces PLY oligomer formation

The serum cholesterol should trigger the next step in pore formation: monomer oligomerization into the pore complex [14], [44], [45], as is observed after cholesterol micelle addition to CDCs [14]. The ability of mouse or human sera to induce PLY oligomer formation was determined using a prepore locked version of PLY (PLY^PPL^), which can oligomerize into the pore complex, but cannot insert the β-barrel pore [46]. A free cysteine was introduced into this mutant at position Asp-117 in order to label the cysteine sulfhydryl with the donor (D; Alexa Fluor 488) or acceptor (A; Alexa Fluor 568) fluorophores. Oligomerization brings the D and A fluorophores within the Förster distance (50% energy transfer) for these dyes and results in D emission quenching [45]. Oligomerization was measured by fluorescence resonance energy transfer (FRET) between D and A dye-labeled PLY^PPL^ [47] in the absence or presence of serum.

**Figure 3:**
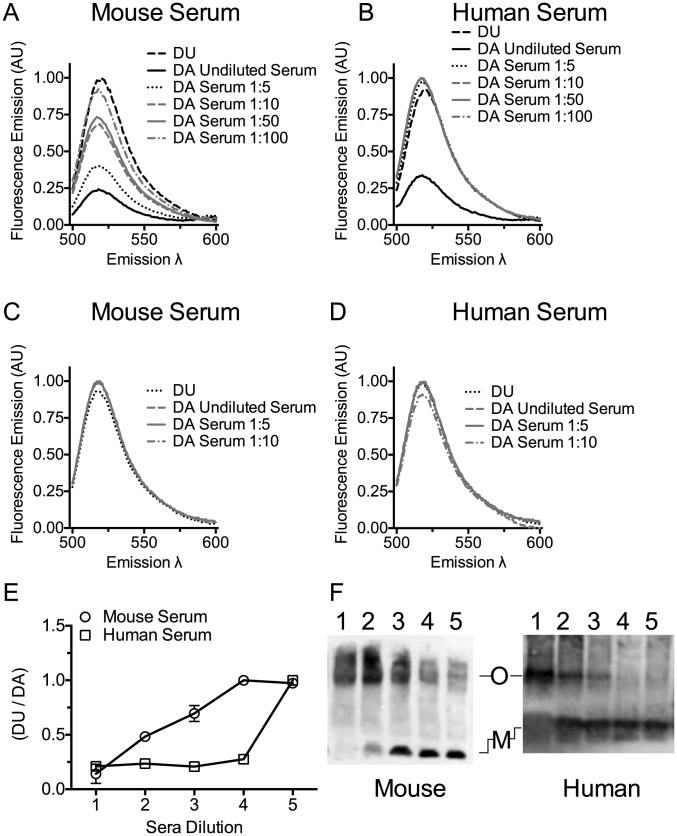
Serum stimulates PLY oligomer formation. Serum stimulated oligomer formation was determined by FRET between donor (D) and acceptor (A) labeled PLY^PPL^ or unlabeled PLY^PPL^ (U). The emission intensity of D in the presence of A at various dilutions of mouse serum (panel A) or human serum (panel B) in the presence of A or U was measured. The decrease in emission intensity of D is dependent on the formation of the oligomer [47]. The ratio of the DU to the DA emission is shown from two independent experiments is shown in panel E. X-axis labels: 1, undiluted serum; 2, 1∶5 dilution; 3, 1∶10 dilution; 4, 1: 50 dilution; 5, 1∶100 dilution. FRET measurements were done as in A and B using PLY^CRM^ as a negative control for any non-oligomer dependent change in the emission. The emission intensity of D labeled PLY^CRM^ in the presence of A labeled PLY^CRM^ is shown only for the 3 least diluted serum samples used in the experiments shown in panels A and B for mouse (C) or human serum (D) dilutions. For each FRET experiment the DA sample was compared to the DU sample (after the subtraction of the appropriate controls from each emission spectrum) to obtain the net change in fluorescence and account for any interference of the sera on the emission. All data were normalized to 1 for comparison. Results are representative of two independent experiments. (F) An immunoblot of an SDS-AGE gel probed with PLY antibody showing the formation of PLY oligomers when PLY^PPL^ was incubated with various dilutions of mouse or human sera. Lanes 1–5 for both panels contain 1 µg of PLY^PPL^ incubated with sera diluted 1∶2, 1∶5, 1∶10, 1∶20 and 1∶40 in a final volume of 50 µl. M, monomer; O, oligomer. The immunoblot is representative of 3 independent experiments.

Mouse serum induced oligomer formation, as determined by FRET, at the highest concentration of serum tested and exhibited a dose-dependent loss of FRET as the mouse serum was diluted (Figure 3A and E). Human serum induced oligomer formation only at the highest serum concentration tested (Figure 3B and E). We also show that both human and mouse sera triggered formation of the SDS-resistant oligomeric complex of PLY in a dose-dependent manner (Figure 3F). A control using the non-binding PLY^CRM^ mutant was not induced to oligomerize by either mouse or human sera (Figure 3C and D).

### Cholesterol carried by ApoB-100 particles of mouse and human sera neutralizes PLY lytic activity

The above studies clearly show serum cholesterol is recognized and bound by PLY, which triggers PLY oligomerization thereby inactivating the toxin. However, cholesterol is carried within serum by several transport lipoproteins. Therefore, is the cholesterol across the spectrum of lipoproteins equally inhibitory or is the inhibitory cholesterol localized to a specific lipoprotein subset? The major cholesterol carriers in mammals are particles containing apolipoprotein ApoA1 (HDL, chylomicrons) and particles containing apolipoprotein ApoB-100 (the major LDL, IDL and VLDL lipoprotein component). To determine if one or more major cholesterol carrier(s) were responsible for the inhibitory activity we separated the fraction of ApoB-100 particles from mouse and human sera (the latter was first depleted of PLY antibodies) using established methods that employ specific precipitation of ApoB-100 particles (primarily LDL/VLDL) with polyethylene glycol (PEG), leaving ApoA1-containing particles in the soluble fraction (primarily HDL) [48].

**Figure 4:**
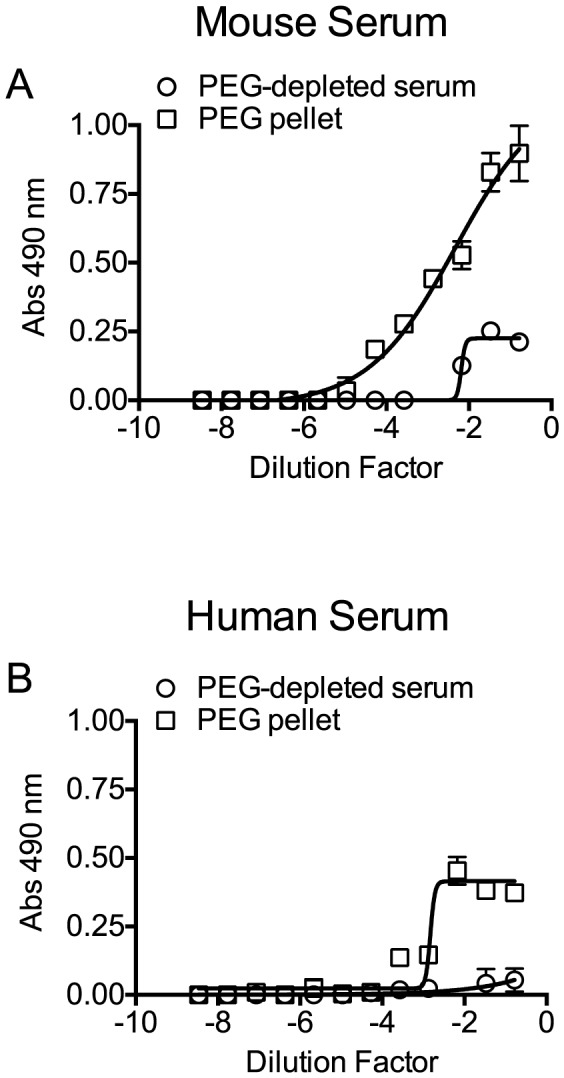
Precipitation of serum ApoB-100-containing particles with PEG. Mouse and human sera particles containing ApoB-100 were precipitated with PEG, and then 96-well microtiter plates were coated with titrated ApoB-100 particle-depleted supernatant or PBS containing the resuspended ApoB-100 particle pellet. Plates were washed and blocked, and then ApoB-100 antibodies were used to detect ApoB-100 containing particles in mouse (A) or human sera (B). Assays were done in duplicate; the data shown represents three independent experiments.

Precipitation efficiency of the ApoB-100 particles was determined by probing PEG-depleted sera (supernatant) and the resulting pellet (suspended in PBS to the same volume as the supernatant) for the presence of ApoB-100 and ApoA1 particles by ELISA assay using an antibody to each specific lipoprotein. The ApoB-100 fraction was efficiently removed from mouse and human sera (Figure 4), with only a small residual amount remaining in the serum following PEG-depletion. The precipitated mouse and human ApoB-100 pellet contained less than 5% of the total HDL (data not shown).

PLY was then incubated with PBS, untreated serum, ApoB-100 particle-depleted serum or precipitated ApoB-100 particles for 20 minutes at 37°C and hemolytic activity determined. Human serum exhibits very low inhibitory activity towards PLY once the PLY antibodies are removed (Table 3): we did not observe a significant difference in the ability of untreated human serum, ApoB-100 depleted serum or the precipitated ApoB-100 particles to inhibit PLY hemolytic activity (Table 4, left columns). In stark contrast the mouse ApoB-100 particle-depleted serum retained virtually no inhibitory activity, whereas the precipitated ApoB-100 particles contained nearly all of the PLY inhibitory activity (Table 4, left columns). These data strongly suggest that cholesterol carried by the mouse ApoB-100 (CH-ApoB-100) is the primary inhibitor of PLY.

**Table 4:** Inhibitory activity and cholesterol content of mouse and human ApoB-100 sera fractions.

Sample	EC_50_ (nM)	EC_50_ serum/PBS	C (µM)	CE (µM)
PBS	0.06±0.03			
Human serum (untreated)	0.9±0.4	15		
ApoB-100-depleted serum	1±0.1	17	32±1	384±4
ApoB-100 pellet	2±0.8	33	196±11	532±10
PBS	0.4±0.09			
Mouse serum (untreated)	**920±20**	**2300**		
ApoB-100-depleted serum	3±2	8	59±6	439±22
ApoB-100 pellet	**880±50**	**2200**	72±4	241±6

Left columns: Shown are the average EC_50_ values, SD and fold-change in EC_50_ values for PLY treated with mouse or human sera (human serum was depleted of PLY antibodies), ApoB-100-depleted serum, ApoB-100 pellet (resuspended in PBS) or PBS alone. Results from 3 independent assays are shown. Right columns: Average cholesterol concentrations and SD for human and mouse sera after ApoB-100-depletion and the ApoB-100 pellet. The PEG precipitated ApoB-100 particles were resuspended to the same volume as the original serum sample for comparative purposes. C, cholesterol. CE, cholesterol esters. Results from 2 independent assays are shown.

### PLY binds specifically to cholesterol carried by ApoB-100 lipoproteins

**Figure 5:**
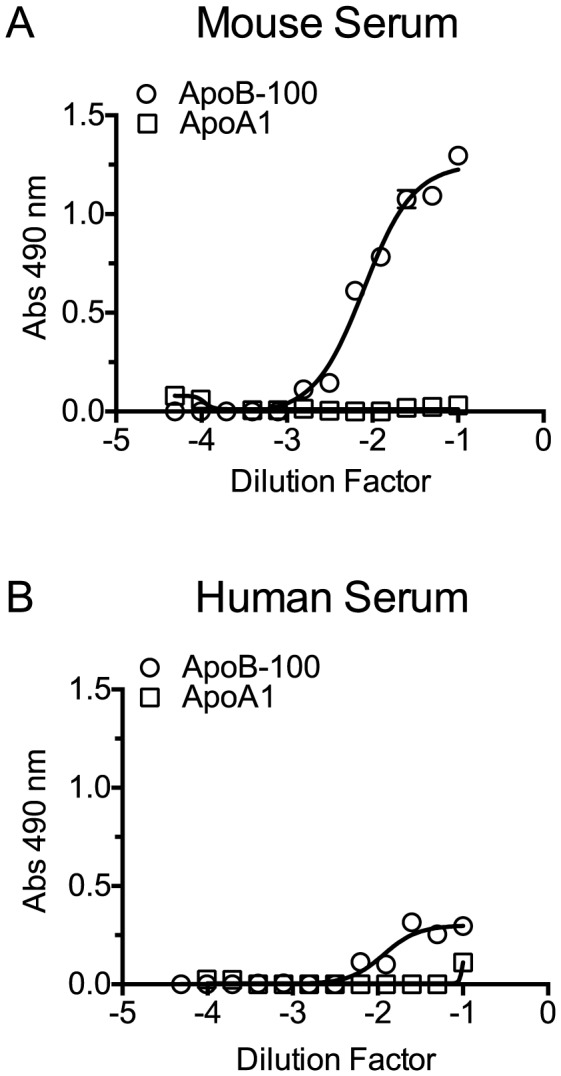
PLY binds to cholesterol carried by ApoB-100 particles in mouse and human sera. PLY^PPL^ was incubated with mouse or human sera for 20 minutes at 37°C to allow it to bind to the lipoprotein cholesterol. The PLY^PPL^-treated sera mixture were serially diluted, added to PLY antibody coated plates and incubated for 2 hours. Plates were washed, and then the PLY^PPL-^mediated capture of lipoproteins containing ApoB-100 and/or ApoA1 from mouse (A) or human sera (B) was detected using ApoB-100 or ApoA1-specific antibodies. Assays were done in duplicate in two independent experiments.

To confirm that PLY preferentially binds human and mouse CH-ApoB-100 a capture ELISA assay was performed with PLY. ELISA plates were coated with PLY antibodies to act as the immobilized capture matrix for PLY. Mouse and human sera were incubated with PLY^PPL^ rather than PLY to minimize any potential lipoprotein particle disruption that might occur with formation of the β-barrel pore by functional PLY. PLY^PPL^-treated sera were then serially diluted in PBS and transferred to the anti-PLY coated microtiter plates to capture the PLY^PPL^. Association of cholesterol carried by either ApoA1 or ApoB-100 lipoproteins with PLY^PPL^ was then detected by probing the captured PLY^PPL^ with ApoB-100 or ApoA1 antibodies to detect VLDL, IDL and LDL lipoproteins, or HDL and chylomicrons, respectively. ApoB-100, but not ApoA1, particles were captured and detected from both sera in this assay (Figure 5). These findings are consistent with the above results and show that PLY interacts with and its activity is inhibited primarily by CH-ApoB-100.

### Cholesterol content of human and mouse ApoB-100 particles

The above results show that the CH-ApoB-100 is responsible for the inhibition of PLY. We therefore also examined these particles for their content of cholesterol with a free 3β-hydroxyl (C) versus cholesterol ester (CE) to determine if the mouse ApoB-100 particles carried a significantly greater amount of C versus CE than did human particles. As is shown in Table 4 (right columns) human ApoB-100 particles contain nearly 3 times as much C as does the mouse analog, which is consistent with previous observations that the majority of serum cholesterol in humans is carried by ApoB-100 particles whereas in mice this is reversed [20]. Yet, we showed above that the cholesterol carried by the mouse ApoB-100 particles is a significantly more potent inhibitor of PLY pore-forming activity than the cholesterol carried by human ApoB-100 particles. These data show that the absolute concentration of cholesterol carried by the respective human and mouse particles is not directly related to their inhibitory activity. As discussed below, these data strongly suggest that the difference in the presentation of cholesterol at the surface of the mouse versus human (and guinea pig) ApoB-100 particles is the basis for the mouse CH-ApoB-100 to function as a potent inhibitor of PLY.

## Discussion

These studies show the sera from mice contain a potent innate inhibitor of the pore-forming activity of multiple CDCs, whereas this inhibitory activity is nearly absent from human and guinea pig sera. Cholesterol that is transported by mouse lipoprotein particles containing ApoB-100 is responsible for inhibiting the pore-forming activity of PLY by stimulating the premature formation of oligomeric complexes. The level of inhibitory activity exhibited by mouse CH-ApoB-100 exceeds that typically observed for the neutralizing capacity of anti-PLY immune sera [37], [39], [49]. Although we measured the inhibitory activity of serum CH-ApoB-100, the potential to inhibit the lytic activities of PLY and other CDCs is not restricted to the circulatory system. ApoB-100 particles function in cholesterol transport to cells where the particle is endocytosed following binding to the LDL receptor [50]. Lipoprotein concentration in the interstitial cellular space is typically 20–30% of the total found in the serum (reviewed in [51]) whereas lipoprotein levels in the sheep lung have been shown to be near 50% of that in the serum [52]. These results have broad implications for the interpretation of studies assessing the contribution of PLY (and other CDCs) to *S. pneumoniae* disease in mouse models and its potential as a vaccine and the extrapolation of these results to human infections. Although there may be several reasons that mice are not normally colonized by *S. pneumoniae* and do not normally develop *S. pneumoniae* diseases [54], [55] PLY inhibition by CH-ApoB-100 may contribute to this natural resistance.

The inhibition of PLY and other CDCs by cholesterol micelles is mediated by the CDC CRM, which recognizes and binds the cholesterol 3β-hydroxyl group [16], [18], [56]. This interaction initiates subsequent conformational changes that lead to formation of the β-barrel membrane pore (reviewed in [7]). Most of the cholesterol carried by the ApoA1 and ApoB-100 particles cannot interact with and inhibit CDCs, as this cholesterol is esterified at the 3β-hydroxyl group and/or is sequestered in the particle core. However, both lipoprotein families contain the majority of their unesterified cholesterol in their outer monolayer shell (reviewed in [19]), [57]–[59] (Figure 1). Results herein show mouse CH-ApoB-100 is recognized and bound by PLY and other CDCs, thereby inactivating them by causing premature assembly of the pore complex. Furthermore, the ability to nearly completely eliminate the inhibitory activity of mouse CH-ApoB-100 by enzymatic oxidation of the cholesterol 3β-hydroxyl shows the cholesterol that inhibits PLY is exposed on the surface of the ApoB-100 particles and is accessible to both cholesterol oxidase and PLY.

In stark contrast to mice, the cholesterol carried by the human cholesterol transport particles exhibited little capacity to inhibit PLY. This difference is remarkable since the majority of the cholesterol in humans is present in ApoB-100 lipoproteins compared to the mouse where the majority is present in the ApoA1 lipoproteins [20]. Cholesterol constitutes 40–50% of the total outer shell lipid in human LDL [60], which is well within the range needed by CDCs for pore formation [15]. Therefore, what is the explanation for the dramatic difference in the capacity of the CH-ApoB-100 in human and mouse particles to inhibit PLY? Although we cannot know with certainty, our results suggest that it is likely to be linked to the lipid environment of the cholesterol and its presentation at the surface of the ApoB-100 particles. The phospholipid fatty acyl chains vary significantly in LDL [60], which affects their ability to pack with cholesterol. We and others have shown the lipid environment surrounding cholesterol heavily influences membrane cholesterol binding by CDCs: the degree of acyl chain saturation and the size of the phospholipid head can significantly impact the ability of CDCs to access and bind to cholesterol [15]–[17], [61]. Hence, the vast difference in the ability of human and mouse CH-ApoB-100 to inhibit PLY may be explained by differences in the exposure of the cholesterol 3β-hydroxyl group on the outer shell surface of the ApoB-100 particles, which arises from variations in the lipid structure and composition of the ApoB-100 outer monolayer between these two species. This may also explain why cholesterol carried by ApoA1 particles does not appear to have any significant inhibitory activity.

Since *S. pneumoniae* has evolved with humans it is possible that the structure of the PLY binding domain has also adapted to resist binding, and therefore inactivation, by human ApoB-100 lipoprotein cholesterol, but not that of the mouse. CDC membrane binding is a composite of cholesterol recognition by the CRM [4] and the subsequent insertion of nearby loops [43], [62]–[64]. The structure of these loops also affects membrane binding [4], [65], which is likely influenced by the lipid environment of the cholesterol [65]. Hence, the evolution of the PLY membrane-binding interface could also contribute to PLY sensitivity to mouse CH-ApoB-100 and its insensitivity to the human and guinea pig analogs. Variation in these loop structures may also explain why we observed that the different CDCs do not exhibit the same susceptibility to the inhibitory activity of the mouse CH-ApoB-100, even though they all bind cholesterol with an identical CRM.

It is interesting to note the serum cholesterol carried by guinea pig lipoproteins, like that of humans, exhibited little ability to inhibit PLY. We also found neutralizing antibodies to PLY in sera from guinea pigs, which, similarly to human serum, accounted for most of the PLY inhibitory activity. These data, though limited, suggest that the guinea pig is naturally susceptible to infection and/or colonization by pneumolysin-expressing streptococcal species whereas mice are not typically naturally colonized or infected by *S. pneumoniae*. It has been shown that pneumonia is the most common guinea pig infection and that *S. pneumoniae* is the most common cause of this pneumonia [66]. In one study *S. pneumoniae* serotype 19F was exclusively present in guinea pigs, which is the most frequent isolate in children age 1-5 years [67]. Therefore, the guinea pig may be an attractive alternative to the mouse model system to test vaccines based on CDCs.

There is no question that many studies have shown a role for PLY in the pathogenesis of *S. pneumoniae* in the mouse (reviewed in [25]). Furthermore, PLY has effects at a distance, as a native PLY-expressing strain of *S. pneumoniae* supports growth of a PLY-deficient strain when mice are co-infected with these two strains [53]. It remains unclear, however, to what extent the mouse LDL inhibitory activity could impact the results on studies of *S. pneumoniae* pathogenesis, or what effect the presence of LDL induced PLY oligomers might have in the mouse model system. It is interesting that retrospective studies in humans have shown that high maternal anti-PLY antibodies are negatively correlated with colonization of infants in at-risk populations [68], [69], yet in the mouse model immunization with PLY does not appear to play a significant role in preventing carriage [70]. These studies suggest that there are unresolved questions between human and mouse *S. pneumoniae* infections, which could be partially due to the differences in the inhibitory capacity of their CH-ApoB-100.

In summary, we have demonstrated that cholesterol carried by the mouse, but not human ApoB-100 particles, possesses a potent innate capacity to inhibit PLY and other CDCs. This interaction triggers the premature formation of the PLY oligomeric pore complex, thereby inactivating the toxin. Our results also suggest the basis for this difference is the presentation of the cholesterol on the surface, which is likely due to differences in the lipid environment in the human and mouse ApoB-100 outer lipoprotein shells and/or the structure of the binding domain. Hence, the contribution of PLY to infections in the mouse may be underestimated by comparison to humans since mouse CH-ApoB-100 is a potent innate inhibitor of PLY. These differences may also have implications for the development and testing of *S. pneumoniae* vaccines based on PLY (and other CDCs) in mouse models and the extrapolation of these results to humans.

## Materials and Methods

### Bacterial strains, plasmids and reagents

The gene for pneumolysin was cloned into pET-15b (Novagen) at sites NdeI and BamHI, and codon optimized by GenScript. The native cysteine (Cys-428) was changed to an alanine to facilitate the introduction of site-specific cysteines in order to place various fluorescent probes into PLY. Hence, the cysteine-less PLY mutant will be referred to as PLY in this manuscript. This plasmid was used as the template for amino acid substitutions. A pre-pore locked pneumolysin was generated by introduction of a disulfide between cysteine-substituted Gly-25 and Glu-159 (PLY^PPL^), similar to that previously described for perfringolysin O (PFO) [46]. A PLY derivative was also generated in which the CRM was knocked out by the substitution of aspartate for Leu-460 (PLY^CRM^) [4].

The native genes for streptolysin O (SLO) and PFO were cloned into pTrcHisA (Invitrogen) as described previously [4], [71]. Chemicals and reagents were purchased from Sigma and Bio-Rad. ApoB and ApoA1 antibodies were purchased from Abcam and MyBioSource, respectively. HRP conjugated secondary antibodies specific for human or mouse were purchased from Bio-Rad, and guinea pig specific secondary antibodies conjugated to HRP were from Thermo Scientific. Sheep red blood cells (RBCs) were purchased from Hemostat. Pooled sera from non-Swiss Albino mice, guinea pigs and humans were purchased from Aleken Biologicals. BALB/c and C57BL/6 mice sera were purchased from Innovative Research. CBA/N serum was provided by David Briles lab (UAB). Mouse monoclonal PLY antibodies were generated by GenScript, using PLY^CRM^ as the immunizing antigen. Sera from rabbits immunized with PLY^CRM^ were obtained from Lampire. Cholesterol oxidase (CHOD) was purchased from Sigma-Aldrich, Affigel-15 gel from Bio-Rad, and Amplex Red Cholesterol Assay Kit, Alexa Fluor 488 C_5_ maleimide and Alexa Fluor 568 C_5_ maleimide were purchased from Invitrogen.

### Toxin production and purification

Hexahistidine tagged, signal peptide deficient versions of PLY, PFO and SLO and associated derivatives were expressed in *E. coli* Tuner cells. Cells were grown, and the recombinant proteins purified and stored as described previously [71]. Fluorescently labeled proteins were stored as described [71], in the absence of reducing agent. Prior to use all proteins were centrifuged at 14,000×*g* (Eppendorf, Centrifuge 5417R) to remove any precipitated proteins and assayed for protein concentration using the Bio-Rad protein assay with bovine serum albumin (BSA; Sigma) as a standard.

### Inhibition of CDC hemolytic activity

The hemolytic activity of PLY, PFO and SLO was determined as previously described [72] except that in addition to performing the serial dilution of each toxin in PBS toxins were also serially diluted with either 10% mouse (non-Swiss albino, Aleken Biologicals; C57BL/6 and BALB/c, Innovative Research), 50% human (Aleken Biologicals), or 50% guinea pig (Aleken Biologicals) sera diluted in PBS (mouse serum was diluted to a greater extent as the mouse serum was found to be significantly more inhibitory than either human or guinea pig sera: all dilutions were taken into account when calculating the EC_50_ of the PLY below). The plates were then incubated for 20 minutes at 37°C and the average effective concentration for 50% hemolysis (EC_50_) and standard deviation (SD) determined. Also, washed sheep RBCs were used instead of human RBCs. The fold-change in the inhibitory activity against PLY or other CDCs for each serum was calculated and reported as follows: EC_50_ of the toxin diluted in the sera/EC_50_ of toxin diluted in PBS. In separate experiments any possible effects of complement on RBC lysis was determined by first heat-inactivating the complement in mouse and human sera at 56°C for one hour [73]. The absolute EC_50_ for CDCs on RBCs varies with different RBCs lots, therefore all experimental data is compared to a positive control with native PLY.

### Cholesterol oxidase treatment of serum

Sera were treated with cholesterol oxidase (CHOD) to oxidize the cholesterol 3β-hydroxyl, converting cholesterol to 4-cholesten-3-one, which is not bound by CDCs. CHOD was solubilized in potassium phosphate buffer (50 mM, pH 7.5) to 100 units/mL. Mouse serum, human serum or PBS were incubated for 30 minutes at 37°C with CHOD (33.6 units/10 ml serum or PBS) to oxidize the cholesterol free 3β-hydroxyl group. The remaining inhibitory activity against PLY-mediated hemolysis was then assayed as described above.

### ELISA assays

Antibody titers to PLY in mouse, guinea pig or human sera were detected by ELISA. Microtiter plates (96-well) were coated overnight with PLY (0.1 µg/well) diluted with carbonate coating buffer (30 mM Na_2_CO_3_, 70 mM NaHCO_3_, pH 9.6). The next morning the plates were rinsed with PBST (PBS plus 0.05% Tween-20, pH 7.4) and then blocked at room temperature for one hour with blocking buffer (3% nonfat dry milk in PBS). Serum samples were serially diluted 1∶5, transferred to the PLY coated plate and incubated for two hours at room temperature. Secondary antibodies conjugated to horseradish peroxidase (HRP) specific to mouse, human (1∶10,000) or guinea pig IgG (1∶5000) were then added for 30 minutes at room temperature to detect antibody binding to PLY. The plates were developed by adding the HRP substrate OPD (o-phenylenediamine dihydrochloride, Sigma) in phosphate citrate buffer, with hydrogen peroxide added right before use, as described by the manufacturer (final concentrations: 0.05 M Na_2_HPO_4_, 0.025 M sodium citrate, 0.4 mg/ml OPD, 0.012% H_2_O_2_). Reactions were terminated by adding 3N sulfuric acid. Absorbance at 490 nm was measured using an iMark microplate reader (Bio-Rad). As a positive control, binding of rabbit sera specific for PLY was detected in the same manner (data not shown) as described above. This assay was also used to quantify the removal of PLY antibodies by affinity chromatography from the various sera (described below). Titer is represented herein by the EC_50_, calculated from the titration curve and defined as the sera dilution factor required for 50% max antibody binding.

An ELISA assay was also used to quantify the relative levels of ApoB-100 and ApoA1 particles present in samples that had been treated with PEG to precipitate ApoB-100 particles (described below). Microtiter plates were coated with ApoB-100 particles-depleted serum or the ApoB-100 particle fraction. Plates were incubated with either rabbit anti-ApoB-100 (1∶50) or goat anti-ApoA1 (1∶1000). The unbound antibody was removed by washing the plates 3 times with HBS and binding detected with the appropriate HRP conjugated secondary antibodies and developed as described above.

### Removal of PLY antibodies from human and guinea pig sera

To remove PLY antibodies from human and guinea pig sera, purified PLY was covalently coupled to Affi-Gel-15 (Bio-Rad) according to the manufacturer's instructions. Human or guinea pig sera (diluted 1∶3 with PBS to reduce binding interference caused by serum proteins) were recirculated 3 times over the column and then collected. Serum dilution by passage over the column was factored into the hemolytic assays. Passage over the PLY-Affi-Gel column removed more than 94% of the PLY antibody.

### Antibody neutralization assay

To determine the contribution of PLY antibodies to the neutralization of PLY hemolytic activity purified PLY (38 nmol/150 µl) was serially titrated (1∶2) into wells containing complete human and guinea pig sera samples or sera depleted of PLY antibodies. The plates were incubated for 20 minutes at 37°C to allow for binding. The hemolytic assay was then completed as described above after RBCs addition. The remaining inhibitory activity in the sera depleted of PLY antibodies was attributed to the inhibitory capacity of the cholesterol carried by serum lipoproteins.

### Selective lipoprotein precipitation from serum

To separate ApoB-100 particles from human or mouse sera, mouse serum was diluted with PBS to match the PLY-antibody depleted human serum dilution. The sera were then made 13.3% PEG 6000 (w/v) and incubated for 20 minutes at room temperature. Samples were then centrifuged for 30 minutes at 10,000 rpm (Eppendorf, model 5417R) at 4°C. The supernatant was removed from the pellet, and the pellet was suspended in PBS to the volume of the beginning sample volume. The hemolytic assay was completed as described above, with PLY incubated in untreated serum, ApoB-100-depleted (PEG-treated) serum or the precipitated ApoB-100 (PEG) pellet.

### Toxin modification with fluorescent probes

Specific modification of the sulfhydryl group of cysteine with sulfhydryl-specific fluorescent probes in the cysteine substituted mutants of PLY was carried out as previously described [74].

### SDS gel electrophoresis of serum treated PLY

To determine whether mouse or human serum proteases cleaved PLY, PLY^CRM^ was labeled with Alexa Fluor 488 at cysteine substituted Asp-117, as described above, and then incubated with 10% mouse or 50% human sera diluted with PBS (1.5 µmol PLY^CRM^/175 µl) for 20 minutes at 37°C. Toxin samples were removed at 0, 15, 30, 45 and 60 minutes, immediately heated to 95°C with SDS sample buffer and then separated by SDS-PAGE using 10% polyacrylamide gels (Bio-Rad). The gels were then imaged using a Gel Logic 1500 Imaging System. Densitometry of resulting fluorescent bands was performed using ImageJ software [75].

### Fluorescence resonance energy transfer (FRET)

The oligomerization of PLY^PPL^ or PLY^CRM^ in which the native cysteine was removed was used to evaluate lipoprotein cholesterol-stimulated oligomer formation. Aspartate 117 was converted to a cysteine in each protein in order to provide a specific site for modification with sulfhydryl-specific probes. Purified PLY or PLY^CRM^ were labeled with donor (D) Alexa Fluor 488 or acceptor (A) Alexa Fluor 568 dyes at Cys-117. FRET analysis of oligomerization was carried out as previously described [76] with the following changes: instead of adding liposomes to stimulate oligomerization the D and A samples were incubated with mouse or human sera (final volume 160 µl, final serum dilutions 1∶3.2, 1∶16, 1∶32, 1∶160, 1∶320) for 20 minutes at 37°C before performing an emission scan of the Alexa Fluor 488 between 500 and 600 nm. The excitation wavelength was set at 470 nm and the bandpass was set at 4 nm.

### Detection of oligomer formation by SDS-agarose gel electrophoresis

SDS-AGE analysis of oligomer formation by PLY^PPL^ was performed as described previously [77], with the following modifications. PLY^PPL^ (19 nmol/50 µl) was incubated with mouse or human sera diluted with HEPES buffered saline (1∶2, 1∶5, 1∶10, 1∶20 and 1∶40 in 200 µl total). The toxin and sera were incubated at 37°C for 20 minutes, then solubilized in SDS sample buffer. Samples were separated by SDS-AGE (1.5%) for 40 minutes at 120 volts. For PLY^PPL^ detection, the proteins were transferred to nitrocellulose membranes and then probed with rabbit anti-PLY sera and rabbit specific HRP-conjugated secondary antibodies. The bands were visualized with chemiluminescence using ECL Western Blotting Detection Reagents (GE Healthcare) and autoradiography.

### Capture ELISA to detect PLY association with cholesterol-containing lipoprotein particles

A capture ELISA was developed to determine if PLY bound to cholesterol in ApoA1 or ApoB-100 lipoproteins. Microtiter plates (96-well) were coated with a mixture of four mouse monoclonal antibodies specific for PLY (diluted 1∶150 in carbonate coating buffer and tested for PLY recognition prior to use) overnight at 4°C, then incubated with blocking buffer (described above) for 1 hour at room temperature. PLY^PPL^ was incubated with mouse or human sera for 20 minutes at 37°C. The PLY^PPL^ serum mixtures were serially diluted in PBS, and then transferred to the antibody-coated plates for 2 hours at room temperature to capture the PLY^PPL^. Unbound proteins were removed by a series of 3 rinses and then the plates were incubated with either rabbit anti-ApoB-100 (1∶50) or goat anti-ApoA1 (1∶1000), both of which cross-react with the human and mouse lipoproteins. After another series of rinses to remove the primary antibody the plates were incubated with the appropriate secondary antibody conjugated to HRP. Plates were then developed as described above for the ELISA analyses.

### Cholesterol quantification in sera samples

Cholesterol concentration in human or mouse sera was determined using Amplex Red Cholesterol Assay Kit following the manufacturer's protocol. The concentration of cholesterol with a free 3β-hydroxyl group was first determined and then total cholesterol (cholesterol + cholesterol ester) was determined after converting cholesterol esters to cholesterol with cholesterol esterase.

## Supporting Information

Figure S1 **Removal of PLY antibodies from human and guinea pig sera.** To remove PLY antibodies from human and guinea pig sera, purified PLY was covalently coupled to Affi-Gel-15. Human or guinea pig sera (initially diluted 1∶3 with PBS) were circulated 3 times over the column and then collected. To determine efficiency of PLY antibody removal from human (A) or guinea pig sera (B) ELISA plates were coated with PLY and then the untreated sera samples or the PLY-Affi-Gel treated sera were titrated and added to the PLY coated plates. The presence of PLY antibodies were then detected with the appropriate anti-IgG. Results are from two independent affinity PLY antibody depletion experiments for each sera sample.
(EPS)

Figure S2 **Proteolytic activity of serum on PLY.** Alexa Fluor 488 labeled PLY^CRM^ was incubated with mouse or human sera for 1 hour, and samples were collected at the times shown. Samples were analyzed by SDS-PAGE, and fluorescence was detected using a Gel Logic 1500 Imaging System. (A) Lanes 1–5: mouse serum + PLY^CRM^. Lanes 6–10: human serum + PLY^CRM^. Densitometric analysis of the PLY in each gel lane was carried out using ImageJ [75] for mouse (B) and human sera (C). The gel and graphs are representative of three independent experiments.
(EPS)

Table S1 **PLY antibody titers in human, guinea pig and mouse sera.** PLY antibody titers were determined for human, guinea pig and mouse sera by coating ELISA plates with PLY then adding titrated sera samples. PLY antibodies were then detected by probing with species-specific IgG secondary antibodies. The titer is represented herein by the EC_50_, defined as the sera dilution factor required for 50% maximum antibody binding as determined by a nonlinear fit of the data. The average titer and SD from at least two batches of pooled sera are shown for human and guinea pig sera. *Note that the level of anti-PLY can vary significantly in various lots of sera from humans and guinea pigs.* PLY antibody titers were determined for one to three pooled batches of sera from the various mouse strains tested. PLY antibody was not detected in the pooled serum from any mouse strain herein. ND, no detectable binding.
(DOCX)
