# Supplementary material for: Mouse, but Not Human, ApoB-100 Lipoprotein Cholesterol Is a Potent Innate Inhibitor of Streptococcus pneumoniae Pneumolysin
Source: PLoS Pathog. 2014 Sep 4;10(9):e1004353. doi: 10.1371/journal.ppat.1004353 (PMC4154877; doi:10.1371/journal.ppat.1004353)
Supplement: Table S1 — PLY antibody titers in human, guinea pig and mouse sera. PLY antibody titers were determined for human, guinea pig and mouse sera by coating ELISA plates with PLY then adding titrated sera samples. PLY antibodies were then detected by probing with species-specific IgG secondary antibodies. The titer is represented herein by the EC50, defined as the sera dilution factor required for 50% maximum antibody binding as determined by a nonlinear fit of the data. The average titer and SD from at least two batches of pooled sera are shown for human and guinea pig sera. Note that the level of anti-PLY can vary significantly in various lots of sera from humans and guinea pigs. PLY antibody titers were determined for one to three pooled batches of sera from the various mouse strains tested. PLY antibody was not detected in the pooled serum from any mouse strain herein. ND, no detectable binding. (DOCX) [file ppat.1004353.s003.docx]

| **Host** | **Titer (1/EC_50_)** |
| --- | --- |
| Human | 5390 ± 4527 |
| Guinea pig | 438 ± 229 |
| Mouse (all strains) | ND |
